# A Comprehensive, Flexible Collection of SARS-CoV-2 Coding Regions

**DOI:** 10.1534/g3.120.401554

**Published:** 2020-08-06

**Authors:** Dae-Kyum Kim, Jennifer J. Knapp, Da Kuang, Aditya Chawla, Patricia Cassonnet, Hunsang Lee, Dayag Sheykhkarimli, Payman Samavarchi-Tehrani, Hala Abdouni, Ashyad Rayhan, Roujia Li, Oxana Pogoutse, Étienne Coyaud, Sylvie van der Werf, Caroline Demeret, Anne-Claude Gingras, Mikko Taipale, Brian Raught, Yves Jacob, Frederick P. Roth

**Affiliations:** * Donnelly Centre, University of Toronto, Toronto, Ontario, Canada; † Department of Molecular Genetics, University of Toronto, Toronto, Ontario, Canada; ‡ Lunenfeld-Tanenbaum Research Institute, Sinai Health System, Toronto, Ontario, Canada; § Unité de Génétique Moléculaire des Virus à ARN, Département Virologie, Institut Pasteur, Paris, France; ** UMR3569, Centre National de la Recherche Scientifique, Paris, France; †† Université de Paris, Paris, France; ‡‡ Univ. Lille, Inserm, CHU Lille, U1192 - Protéomique Réponse Inflammatoire Spectrométrie de Masse - PRISM, F-59000 Lille, France; §§ Molecular Architecture of Life Program, Canadian Institute for Advanced Research, Toronto, Ontario, Canada; *** Department of Medical Biophysics, Princess Margaret Cancer Centre, University of Toronto, Toronto, Ontario, Canada; ††† Department of Computer Science, University of Toronto, Toronto, Ontario, Canada

**Keywords:** SARS-CoV-2, coding sequence collection, Gateway-compatible, TEV (tobacco etch virus) sequence

## Abstract

The world is facing a global pandemic of COVID-19 caused by the SARS-CoV-2 coronavirus. Here we describe a collection of codon-optimized coding sequences for SARS-CoV-2 cloned into Gateway-compatible entry vectors, which enable rapid transfer into a variety of expression and tagging vectors. The collection is freely available. We hope that widespread availability of this SARS-CoV-2 resource will enable many subsequent molecular studies to better understand the viral life cycle and how to block it.

A global pandemic of the coronavirus disease COVID-19, a severe respiratory illness caused by a novel virus from the family *Coronaviridae* (SARS-CoV-2), has infected millions and caused hundreds of thousands of deaths (World Health Organization 2020a). COVID-19 manifestation in patients can range from a lack of symptoms to severe pneumonia and death (Huang *et al.* 2020). Person-to-person spread through respiratory droplets has been identified as a major source of transmission of the virus (Yu *et al.* 2020). Various measures, from social distancing to nationwide lockdowns, have been imposed to contain and control the transmission of SARS-CoV-2 (Cohen and Kupferschmidt 2020). Despite these measures, the number of confirmed COVID-19 cases has continued to rise (World Health Organization 2020a), highlighting the need for an effective vaccine and antiviral agents. Furthermore, the extrapolations concerning the evolution of the pandemic are particularly alarming (Ferguson *et al.* 2020). It is therefore of intense and pressing interest to better understand this virus and its interaction with host cells on a molecular level.

Shortly after the outbreak, the complete genome of two SARS-CoV-2 strains were published (Chan *et al.* 2020; Wu *et al.* 2020). Using the genome sequence as a reference, Chan *et al.* identified 12 viral open reading frames (ORFs), including one encoding ORF1AB, a large polyprotein which is post-translationally processed into 16 proteins (Chan *et al.* 2020). More recently, Wu *et al.* discovered two additional viral ORFs (*ORF9Bwu* and *ORF10wu*) with unclear functions (Wu *et al.* 2020). Progress on molecular characterization has been made on several viral proteins (Walls *et al.* 2020; Zhang *et al.* 2020), providing valuable insights into host-virus interaction, but more research is necessary. The Gateway system offers efficient and high-throughput transfer of the viral coding sequences (CDSs) into a large selection of Gateway-compatible destination vectors used for protein expression in many biological systems, *e.g.*, *Escherichia coli*, *Saccharomyces cerevisiae*, insect, or mammalian cells (Walhout *et al.* 2000). Broad availability of a collection of SARS-CoV-2 CDSs has the potential to enable many downstream biochemical and structural studies and thus a better understanding of processes within the viral life cycle, including scalable assays for screening drug candidates that could potentially disrupt these processes.

## Materials and Methods

### Synthesis of viral coding sequences

Based on the published annotation of the genome sequence of the HKU-SZ-005b (GenBank MN975262; Chan *et al.* 2020) and Wuhan-Hu-1 (GenBank MN908947; Wu *et al.* 2020) isolates of SARS-CoV-2, we requested the synthesis of viral coding sequences (GenScript and Integrated DNA Technologies), including termination codons and *attB* recombination sequences, with optimization of codon usage to reduce GC content and optimize expression in human and insect cells. A start codon was added to *NSP2–16* to allow independent transcription and translation, as the endogenous products are derived from *ORF1AB* by post-translational processing. *ORF9Bwu*, an alternative ORF within the *N* gene from SARS-COV-2 (Wu *et al.* 2020), was subsequently amplified by polymerase chain reaction (PCR) from the viral *N* gene with primers listed in Table S1.

### Generation of Gateway-compatible viral coding sequence clone collections

Synthesized viral coding sequences were incorporated into Gateway Entry plasmids: either pDONR207 (Invitrogen Cat #12213013) or pDONR223 (Rual *et al.* 2004). To enable C-terminal fusion constructs, we also generated an equivalent set of Gateway-compatible clones without termination codons. These clones were made by either PCR-amplifying the whole plasmid with primers that eliminated the stop codon, or by amplifying CDS regions from the first collection, using downstream primers with complementary regions that were internal to each stop codon, and which simultaneously incorporated the flanking sequences necessary for incorporation into a Gateway Entry plasmid [pDONR207, pDONR221 (Invitrogen Cat #12536017) or pDONR223].

Expression clones with N-terminal fusion tags (*e.g.*, for purification) can be produced simply by preparing the appropriate Gateway-compatible Destination vector. However, to enable the subsequent removal of such N-terminal fusion tags, we generated an additional set of clones containing, at the N-terminus of the ORF, a recognition sequence for nuclear inclusion protease from tobacco etch virus (TEV). TEV sequences were incorporated by amplifying CDS regions from the first collection using forward primers that also provide TEV sequences with the original reverse primers.

Each SARS-CoV-2 CDS bacterial clone (DH5α *E. coli* strain, NEB Cat# C2987) was isolated from a single colony, and its inserted CDS was confirmed by full-length Sanger sequencing (The Centre for Applied Genomics, Toronto, Canada). All clones with a pDONR221 or pDONR223 backbone were sequenced with M13F and M13R primers. Clones with a pDONR207 backbone were sequenced with customized forward and reverse primers. All primer sequences are available in Table S1.

### Data availability

Clones are available through Addgene. Table S1 contains all primers used. Table S2 contains detailed descriptions of clones in the collection and links to the clone resource available from Addgene. Supplemental material available at figshare: https://doi.org/10.25387/g3.12725096.

## Results and Discussion

A total of 98 clones (Table 1) are currently included in the Gateway-compatible collection, covering 28 out of 29 total annotated CDSs in the SARS-CoV-2 genome. *NSP11* was omitted due to the incompatibility of its 36 base pair length with the Gateway cloning system (Cheo *et al.* 2004). All 28 of these CDS regions are available as clones with and without termination codons. The ‘no-stop’ collection was further extended to include six clones encoding different cleaved products of the spike (S) protein — “S-fragment” 1–6. We also included two CDS variants with in-frame deletions (“S-24nt” and “E-27nt”), one truncated CDS variant (“ORF8B-truncated”), that were each detected by recent viral transcriptome mapping efforts (Davidson *et al.* 2020, Kim *et al.* 2020) and two missense catalytic variants (*NSP3* C857A and *NSP5* C146A; Gordon *et al.* 2020).

**Table 1:** The genome-scale SARS-CoV-2 coding sequence clone collection

Gene Symbol	CDS Name	Putative Function/Domain	AA Length	Clone Status		
				STOP	NO STOP	TEV
*ORF1AB*	NSP1	Suppress antiviral host response	180	✓	✓	✓
	NSP2	Unknown	639	✓	✓	✓
	NSP3	Putative PL-pro domain	1,946	✓	✓	✓
	NSP3-Cys857Ala	Putative PL-pro domain (with Cys857Ala variant)	1,946	✓	✓	NA
	NSP4	Complex with NSP3 & 6 for DMV (double-membrane vesicle) formation	501	✓	✓	✓
	NSP5	3CL-pro domain	307	✓	✓	✓
	NSP5-Cys146Ala	3CL-pro domain (with Cys146Ala variant)	307	✓	✓	NA
	NSP6	Complex with NSP 3 & 4 for DMV formation	291	✓	✓	✓
	NSP7	DNA primase subunit	84	✓	✓	✓
	NSP8	DNA primase subunit	199	✓	✓	✓
	NSP9	RNA/DNA binding activity	114	✓	✓	✓
	NSP10	Complex with NSP14: Replication fidelity	140	✓	✓	✓
	NSP12	RNA-dependent RNA polymerase	919	✓	✓	✓
	NSP13	Helicase	602	✓	✓	✓
	NSP14	ExoN: 3′-5′ exonuclease	528	✓	✓	✓
	NSP15	XendoU: poly(U)-specific endoribonuclease	347	✓	✓	✓
	NSP16	2’-O’-MT: 2’-O-ribo methyltransferase	299	✓	✓	✓
*S*	S	Spike glycoprotein trimer that binds to host cell receptors (*e.g.*, ACE2)	1,273	✓	✓	✓
*S*	S-24nt	Spike glycoprotein trimer (minus 8 amino acids)	1,265	✓	✓	NA
*S*	S-frag1	Entire Ectodomain	1,213	NA	✓	NA
*S*	S-frag2	Entire Ectodomain without the signal peptide	1,199	NA	✓	NA
*S*	S-frag3	N-term fragment after the furin cleavage	686	NA	✓	NA
*S*	S-frag4	N-term fragment after the furin cleavage without the signal peptide	672	NA	✓	NA
*S*	S-frag5	C-terminal Ectodomain from the furin cleavage site	528	NA	✓	NA
*S*	S-frag6	C-terminal Ectodomain from the Tmpress 2 priming site	399	NA	✓	NA
*ORF3A*	3A	Induce inflammatory response and apoptosis	275	✓	✓	✓
*ORF3B*	3B	Induce inflammatory response and inhibit the expression of IFNβ	58	✓	✓	✓
*E*	E	Envelope protein pentamer	75	✓	✓	✓
*E*	E-27nt	Envelope protein pentamer (minus 9 amino acids)	66	✓	✓	NA
*M*	M	Membrane protein	222	✓	✓	✓
*ORF6*	6	Antagonize STAT1 function and IFN signaling, and induce DNA synthesis	61	✓	✓	✓
*ORF7A*	7A	Induce inflammatory response and apoptosis	121	✓	✓	✓
*ORF7B*	7B	Induce inflammatory response	43	✓	✓	✓
*ORF7B*	7B-trunc	Induce inflammatory response (with N terminus truncated)	20	✓	✓	NA
*ORF8*	8	Induce apoptosis and DNA synthesis	121	✓	✓	✓
*N*	N	Facilitate viral RNA packaging	419	✓	✓	✓
*ORF9B*	9B	Induce apoptosis	98	✓	✓	✓
*ORF9Bwu*	9Bwu	Unknown	73	✓	✓	NA
*ORF10wu*	10wu	Unknown	38	✓	✓	NA

✓ indicates that clone is available; NA indicates that the clone was not available the time of this writing.

Although our collection facilitates tagging of SARS-CoV-2 proteins for various functional studies, certain applications require removal of tags at some stage, for example, after protein purification. Fusion proteins can potentially interfere with the yield, structure, and function of purified proteins, such as during large scale production and crystallography studies (Booth *et al.* 2018). To address this we expanded our collection to include clones containing an N-terminal recognition sequence for the nuclear inclusion protease from tobacco etch virus (TEV; Carrington and Dougherty 1987; Carrington and Dougherty 1988). The TEV sequence is one of the best characterized and widely used endoproteolytic reagents due to its stringent sequence specificity, ease of production, and ability to tolerate a variety of residues at the P1’ position of its recognition site (Waugh 2011). We note that our clones are not expression vectors in and of themselves, and we have not yet assessed the expression of any of our clones after moving to a Gateway Destination expression vector. However, we note that our Gateway-compatible collection allows users the flexibility to conveniently move any of the SARS-CoV-2 ORFs into any Gateway Destination expression vector with any preferred N-terminal or C-terminal fusion.

To promote open-access dissemination of the collection, all clones have been deposited to the non-profit organization Addgene (Kamens 2015), and are freely available from the authors under circumstances where Addgene cannot be used. Table S2 summarizes all CDSs in the collection, together with their nucleotide sequences, nucleotide and amino acid lengths and links for ordering clones.

We hope that this SARS-CoV-2 CDS-clone collection will be a valuable resource for many applications, including study of how coronaviruses can exploit host cellular processes for the viral replication cycle (de Wilde *et al.* 2018), understanding virus-host protein-protein interactions (Gordon *et al.* 2020; Lasso *et al.* 2019), production of recombinant virus proteins for structural studies (Edavettal *et al.* 2012), mapping of protein subcellular localization using N-terminal fluorescent reporters (Tanz *et al.* 2013), or development of vaccines or other therapeutics (Jing *et al.* 2012; McDonald *et al.* 2007).
